# *Rickettsia aeschlimannii* Infection, Algeria

**DOI:** 10.3201/eid1411.071221

**Published:** 2008-11

**Authors:** Nora Mokrani, Philippe Parola, Soraya Tebbal, Mokhtar Dalichaouche, Ahmed Aouati, Didier Raoult

**Affiliations:** Clinique des Maladies Infectieuses, Batna, Algeria (N. Mokrani, S. Tebbal); Unité des Rickettsies CNRS-IRD UMR, Marseille, France (P. Parola, D. Raoult); Service des Maladies Infectieuses, Constantine, Algeria (M. Dalichaouche, A. Aouati)

**Keywords:** Rickettsia, Algeria, Africa, ticks, letter

**To the Editor**: Only 2 cases of *Rickettsia aeschlimannii* infection have been reported. We report 2 additional cases documented in Algeria by immunofluorescence (IF) assays and confirmed by Western blot (WB) assays and cross-adsorption studies.

Tick-borne rickettsioses are now recognized as emerging or reemerging human infections worldwide. These zoonoses, caused by intracellular bacteria within spotted fever group (SFG) *Rickettsia* spp., share characteristic clinical features including fever, rash, and sometimes inoculation eschar at the bite site (*1*). In North Africa, cases of rickettsioses are rarely documented (*2*). In Algeria, only Mediterranean spotted fever caused by *R*. *conorii* has been described (*3*).

From 2000 through 2006 in Algeria, all patients with suspected rickettsioses seen at the infectious diseases units of Constantine and Batna hospitals were included in a prospective study; clinical and epidemiologic data and acute-and convalescent-phase serum samples obtained 2–4 weeks later were collected. Serum samples were sent to Marseille, France, where they were analyzed by an IF assay, using 9 SFG rickettsial antigens (*R*. *conorii conorii*, *R. conorii israelensis*, *R*. *africae*, *R. sibirica mongolitimonae*, *R. aeschlimannii*, *R. massiliae*, *R. helvetica*, *R. slovaca*, and *R. felis*) and a typhus group antigen (*R. typhi*) (*3*). The IF assay result was considered positive 1) if immunoglobulin (Ig) G titers were >128 and/or IgM titers were >64 for *R. conorii* and 2) if IgG titers were >64 and/or IgM titers were >32 for other rickettsial antigens (*3*). When cross-reactions between several antigens were noted, rickettsial antigen was considered to represent the infectious agent if titers of IgG and/or IgM antibody against this antigen were at least 2-fold higher than titers of IgG and/or IgM antibody against other rickettsial antigens (*3*,*4*). When the difference in titers among several antigens was lower than 2-fold, WB assays and cross-adsorption studies were performed (*4*,*5*). A total of 135 patients were included in the study. We describe 2 cases of *R. aeschlimannii* infection. Cases caused by other SFG rickettsiae will be reported elsewhere.

An 80-year-old man who reported contact with dogs parasitized by ticks had a 7-day history of high fever, headache, myalgia, and vomiting. On physical examination, a generalized maculopapular rash, 2 eschars (right shoulder and knee), and bilateral hemorrhagic signs on the retina were noticed. Elevated levels of liver enzymes (aspartate aminotransferase 187 U/L, alanine aminotransferase 108 U/L), hyponatremia (sodium 120 mmol/L), and hypokalemia (potassium 2.9 mmol/L) were found. IF assay showed raised levels of IgG/IgM against *R. aeschlimannii* (512/64) and *R. conorii* (128/0).

The second patient, a 36-year-old man, reported a 15-day history of fever with headache and failure of amoxicillin and cotrimoxazole treatments. Oral aphtous, a maculopapular rash, and purpuric lesions on the arms were noticed. IF assay showed raised levels of IgG/IgM at the same titer (2,048/32) against *R. conorii*, *R. aeschlimannii*, and *R. massiliae.* WB assays and cross-adsorption studies confirmed that antibodies were directed against *R. aeschlimannii* (Figure). Both patients recovered after doxycycline treatment (*1*).

**Figure:**
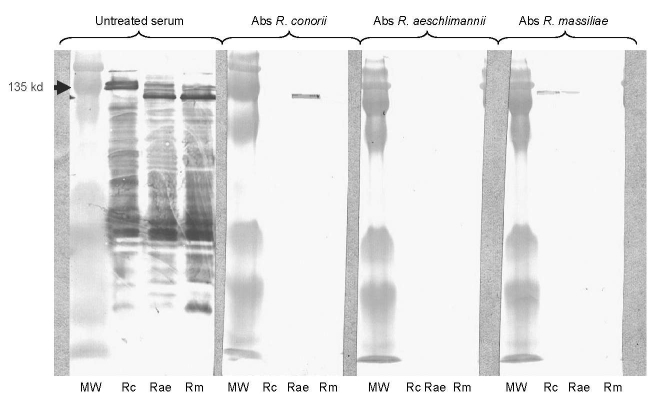
Western blot assay (WB) and cross-adsorption studies in serum of a patient with rickettsiosis in Algeria. Immunofluorescent assay showed raised levels of immunoglobulin (Ig) G/M at the same titer (2,048/32) against *Rickettsia conorii*, *R. aeschlimannii*, and *R. massiliae*. Lanes Rc, Rae, and Rm: WB assay using *R. conorii*, *R. aeschlimannii*, and *R. massiliae* antigens, respectively. MW, molecular weights are indicated on the left. Untreated serum, late serum samples tested by WB. When adsorption is performed with *R. aeschlimannii* antigens, homologous and heterologous antibodies disappear, but when it is performed with *R. conorii* antigens and *R. massiliae,* homologous antibodies disappear but heterologous antibodies persist. This result indicates that antibodies are specifically directed against *R. aeschlimannii*. Abs, absorbed.

*R. aeschlimannii* was first characterized as a new SFG rickettsia after its isolation from *Hyalomma marginatum marginatum* ticks in Morocco in 1997 (*6*). Thereafter, *R. aeschlimannii* has been detected in this tick species in southern Europe and North Africa (*7*), as well as in *H. m. rufipes* in sub-Saharan Africa (*1*). Preliminary data have suggested that these *Hyalomma* organisms may be not only vectors but also reservoirs of *R. aeschlimannii* and as a consequence, the geographic distribution of *R. aeschlimannii* would be at least that of these ticks throughout southern Europe and Africa (*8*).

Although WB assays and cross-adsorption studies are time-consuming and only available in specialized reference laboratories, new data can be obtained for a better understanding of rickettsioses. We have added the description of 2 more cases of infection with *R. aeschlimannii*. Only 2 cases of human infection caused by this rickettsia had been previously reported, including infection in a patient returning to France from Morocco, and another in a patient in South Africa (*9*,*10*).

Clinicians should be aware that several tick-borne rickettsial pathogens are present in Algeria. Specific clinical features may be directly influenced by the *Rickettsia* sp. involved, the rickettsial infection rate of the vector, and tick behavior. *H. marginatum* ticks readily bite humans, and persons may receive multiple simultaneous tick bites. Furthermore, the high infectious rate of these ticks by *R. aeschlimannii* has been reported (*1*). Therefore, the probability of being bitten by several infected *H. marginatum* ticks is high and can lead to several eschars in patients, a characteristic of few tick-borne rickettsioses. Finally, although doxycycline is the reference treatment for rickettsioses, rifampin has been used (*1*). However, although *R. conorii* is susceptible in vitro to this drug, *R. aeschlimannii* is resistant. Because patients suspected of having rickettsiosis must receive prompt presumptive treatment, the presence of *R. aeschlimannii* in Morocco reinforces the need to use doxycycline as a first-line drug.
